# A case of esophageal ulcer and hemorrhage due to aberrant subclavian in a COVID positive patient

**DOI:** 10.1093/jscr/rjab643

**Published:** 2022-01-26

**Authors:** Ahmad Hlayhel, Lindsey Foran, Aakash Trivedi, Jamshed Zuberi, Luis Cerda, John Danks

## Abstract

Coronavirus Disease 2019 (COVID-19), a global pandemic, is a respiratory infection that impairs the lungs among many other organs. We report a case of a COVID-19 positive patient requiring prolonged mechanical ventilation with nasogastric tube for enteral feeding, leading to esophageal ulcer and hemorrhage, from an aberrant right subclavian artery.

## INTRODUCTION

Coronavirus Disease 2019 (COVID-19), a global pandemic, is a respiratory infection that impairs the lungs among many other organs. It is transmitted widely through human-to-human contact in which symptomatic and asymptomatic patients can be the source of spread. Common initial symptoms include fever, cough, fatigue, anosmia, dysgeusia, anorexia and myalgias. Progression of the disease can be accompanied by dyspnea and hypoxemia; a significant feature is the potential for rapid respiratory failure progressing to pneumonia, acute respiratory distress syndrome (ARDS) and even death. We report a case of pneumonia secondary to COVID-19 requiring prolonged mechanical ventilation with nasogastric tube for enteral feeding. As a consequence of prolonged nasogastric tubes (NGT) requirement, the patient had subsequent mucosal damage eroding into an aberrant right subclavian artery. This is only the fourth ever described case of a NGT leading to erosion into an aberrant right subclavian artery, and the first ever likely secondary to an admission for COVID-19.

## CASE PRESENTATION

**Figure 1:**
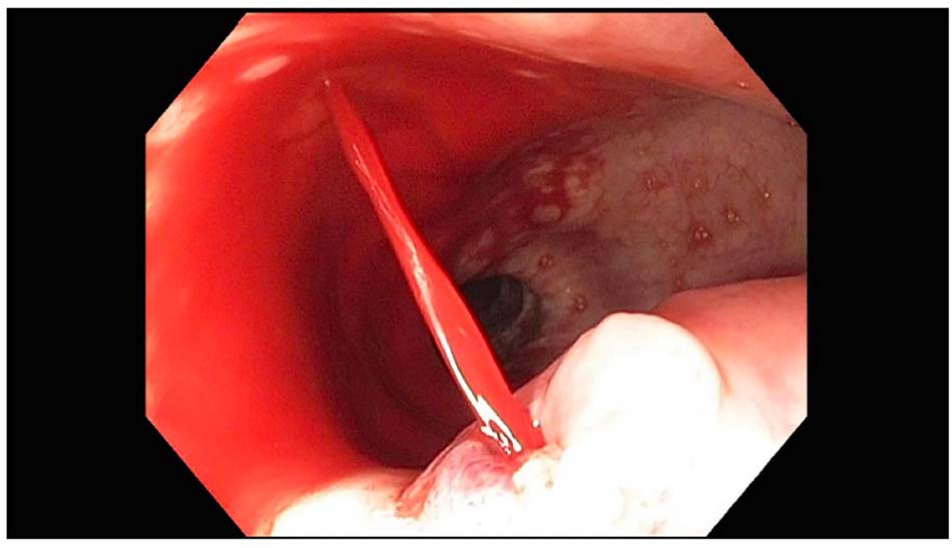
EGD showing actively spurting blood from midesophageal ulcer.

**Figure 2:**
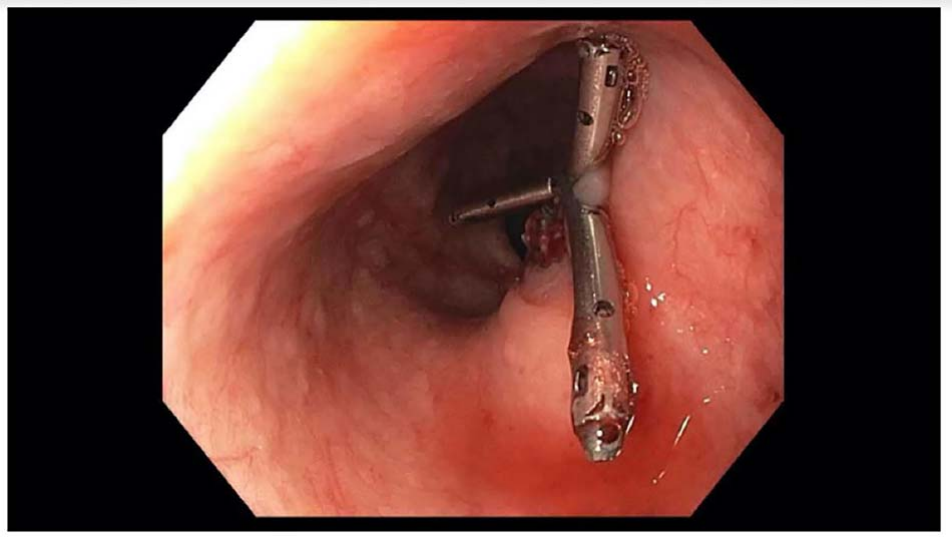
Repeat EGD showing three clips from prior EGD; no evidence of active bleeding.

**Figures 3:**
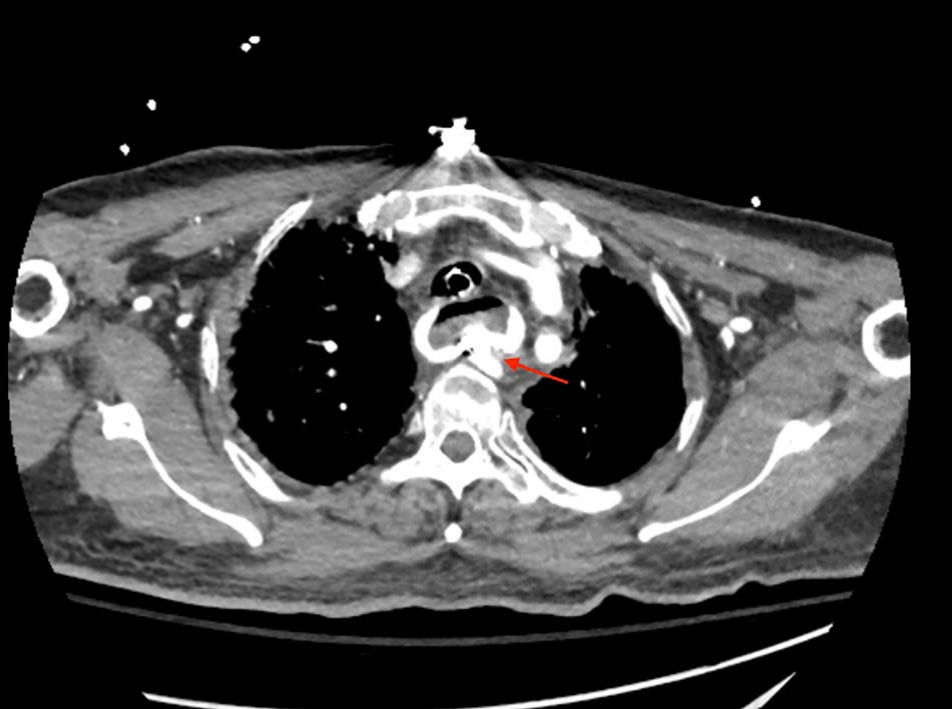
CT thoracic angiogram showing actively bleeding aberrant right subclavian artery perforating into the esophagus (red arrow).

**Figure 4:**
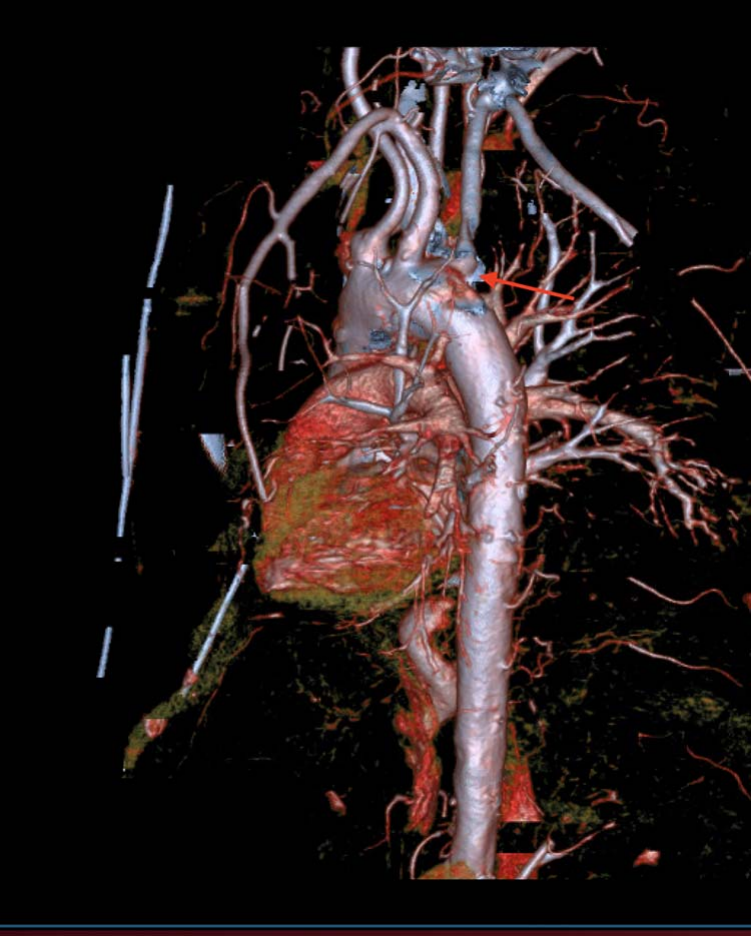
CT thoracic showing the arch of the aorta with the take-off of the right subclavian artery (red arrow) distal to the left subclavian artery.

A 71-year-old male with past medical history of type 2 diabetes and hypertension presented to the emergency department at St. Joseph’s University Medical Center with severe respiratory distress. On nonrebreather, the patient’s oxygen saturation was 49%. The patient was subsequently intubated. Evaluation revealed pneumonia secondary to COVID-19 leading patient to be intubated on ventilator support for 4 weeks. During this time patient was maintained on enteral feeding through nasogastric tube, and was on gastrointestinal prophylaxis Protonix 40 mg daily for the duration of his admission. Ultimately, the patient made full recovery and was sent home; however, approximately 3 weeks later, he presented to the emergency department due to syncope. The patient experienced large-volume bright red hematemesis. He became hypotensive and was transfused three units of packed red blood cells and started on pressors. The patient was intubated and transferred to the intensive care unit (ICU) with acute blood loss anemia and hypovolemic shock. Here, an esophagogastroduodenoscopy (EGD) was performed revealing midesophageal bleed (Fig. 1). Hemostasis was achieved with clipping; however, post-procedurally, the patient again became hypotensive. Repeat EGD showed a nonbleeding esophageal ulcer with three clips in good position (Fig. 2). Computed tomography (CT) angiogram of the thorax was performed revealing an actively bleeding aberrant right subclavian artery perforating into the esophagus (Figs 3 and 4). The right subclavian artery was noted to be of normal caliber without ectasia or aneurism and the esophagus was free of any other pathology. Thoracic and vascular surgery were consulted; however, the bleed was deemed of nonsurvivable pathology due to comorbidities and current clinical status. Ultimately, the family of the patient decided to withdraw care and the patient passed.

## DISCUSSION

NGT are often used in intubated patients for gastric decompression, medication administration or feeding. A myriad of complications can arise with prolonged use such as kinking, impaction, obstruction and perforation. Incorrect placement can penetrate the respiratory tract, or even, rarely, intravascularly or intracranially [1]. A potentially severe complication of NGT use is ulceration of esophageal and gastric mucosa [1, 2]. Perhaps, double-lumen design prevents these ulcers [2]. Yet, cases have been reported of double-lumen nasogastric tubes causing esophageal and gastric ulcers.

Our patient initially presented with COVID-19 pneumonia and was intubated with an NGT for an extended period. On his second admission, he presented with a proximal esophageal ulcer, which may have resulted from prolonged NGT use. It is important for healthcare providers to be aware that, while often necessary, prolonged NGT use can be possibly detrimental to our patients.

Furthermore, literature has revealed that COVID-19 can be a potential cause of ulceration in the gastrointestinal tract. A recent retrospective multicenter study performed by Blackett *et al.* showed a higher proportion of procedures performed in COVID-19 patients found upper gastrointestinal tract ulcers, erosions or esophagitis compared to negative or untested patients. Additionally, active bleeding was found more in positive patients, at 8.3% compared to 3.1% of negative patients and 2.4% of untested patients. In their study, endoscopies done for anemia or bleeding in COVID-19 positive patients required roughly twice the rate of hemostatic clipping, epinephrine injections or electrocoagulation (40.0%) compared to negative (17.1%) or untested patients (23.4%) [3]. The clinical spectrum of COVID-19 is not yet completely understood; however, proposed causes of peptic ulcer disease in COVID-19 patients include infection related stress, direct epithelial damage or cytokine storm related mucosal inflammation [4]. This coupled with the potential for ulceration from continued NGT use due to intubation gives our patient two possible sources of his esophageal ulcer.

The aortic arch, located in the superior mediastinum, has three branches: brachiocephalic artery, left common carotid artery and left subclavian artery. The right subclavian artery, a branch of the brachiocephalic artery, travels laterally between the anterior and middle scalene muscles. However, there have been documented cases of anomalous arteries, including right subclavian, as in our patient. A left aortic arch with an aberrant right subclavian artery is the most common congenital anomaly of the aortic arch with a prevalence of 0.5–2.0%, causing the right subclavian to have an oblique retroesophageal course [5]. Our patient had an aberrant right subclavian artery in proximity to the esophagus. The ulceration, due to either prolonged nasogastric tube insertion or COVID-19, coupled with the aberrant artery proved to be catastrophic for this patient and ultimately led to his demise.

## CONCLUSION

The development of an ulcer in the proximal esophagus led to our patient’s death which may have resulted from prolonged nasogastric tube use. On the other hand, it could have been caused through a wide array of mechanisms secondary to a severe COVID-19 infection. Regardless of the cause, given that patients’ aberrant right subclavian artery coupled with an esophageal ulcer ultimately led to this patient’s death. Moving forward, the presence of an ulcer should be kept in mind when managing COVID-19 positive patients or those with prolonged NGT placement. However, it is difficult to know about anomalous arterial supplies in specific patients. Therefore, this unique patient presentation was difficult to completely understand and treat.

## Data Availability

The authors declare that data supporting the findings of this study are available within the article.
